# Impact of *BCR::ABL1* transcript type on RT-qPCR amplification performance and molecular response to therapy

**DOI:** 10.1038/s41375-022-01612-2

**Published:** 2022-06-08

**Authors:** Matthew Salmon, Helen E. White, Hana Zizkova, Andrea Gottschalk, Eliska Motlova, Nuno Cerveira, Dolors Colomer, Daniel Coriu, Georg N. Franke, Enrico Gottardi, Barbara Izzo, Tomas Jurcek, Thomas Lion, Vivien Schäfer, Claudia Venturi, Paolo Vigneri, Magdalena Zawada, Jan Zuna, Lenka Hovorkova, Jitka Koblihova, Hana Klamova, Marketa Stastna Markova, Dana Srbova, Adela Benesova, Vaclava Polivkova, Daniela Zackova, Jiri Mayer, Ingo Roeder, Ingmar Glauche, Thomas Ernst, Andreas Hochhaus, Katerina Machova Polakova, Nicholas C. P. Cross

**Affiliations:** 1 Faculty of Medicine, University of Southampton, Southampton, UK; 2 Wessex Regional Genetics Laboratory, Salisbury NHS Foundation Trust, Salisbury, UK; 3 Institute of Hematology and Blood Transfusion, Prague, Czech Republic; 4 Institute for Medical Informatics and Biometry (IMB), Carl Gustav Carus Faculty of Medicine, TU Dresden, Dresden, Germany; 5 Portuguese Oncology Institute of Porto, Porto, Portugal; 6 Pathology Department, Hospital Clinic, Institut d’ Investigacions Biomèdiques August Pi i Sunyer (IDIBAPS), CIBERONC, Barcelona, Spain; 7 Fundeni Clinical Institute, Hematology Department, Bucharest, Romania; 8 Hematology Department, Faculty of Medicine, University of Medicine and Pharmacy “Carol Davila”, Bucharest, Romania; 9 University of Leipzig Medical Center, Department for Hematology, Cellular Therapies and Hemostaseology, Leipzig, Germany; 10 Laboratory of Chemical and Clinical Analysis “Area 3” A.O.U San Luigi Gonzaga-Orbassano, Turin, Italy; 11 Department of Molecular Medicine and Medical Biotechnology University ‘Federico II’ and CEINGE - Advanced Biotechnologies, Naples, Italy; 12 Center of Molecular Biology and Gene Therapy, Internal Hematology and Oncology Clinic, Faculty Hospital Brno and Faculty of Medicine, Masaryk University, Brno, Czech Republic; 13 Labdia Labordiagnostik / St. Anna Children´s Cancer Research Institute (CCRI), Vienna, Austria; 14 Abteilung Hämatologie/Onkologie, Klinik für Innere Medizin II, University of Jena, Jena, Germany; 15 IRCSS Azienda Ospedaliero-Universitaria di Bologna, Istituto di Ematologia “Seràgnoli”, Bologna, Italy; 16 University of Catania, Department of Clinical and Experimental Medicine, Center of Experimental Oncology and Hematology, Catania, Italy; 17 The University Hospital in Krakow, Krakow, Poland; 18 CLIP, Dept. of Paediatric Haematology and Oncology, Second Faculty of Medicine, Charles University and University Hospital Motol, Prague, Czech Republic; 19 Internal Hematology and Oncology Clinic, Faculty Hospital Brno and Faculty of Medicine, Masaryk University, Brno, Czech Republic; 20 National Center for Tumor Diseases (NCT), Dresden, Germany: German Cancer Research Center (DKFZ), Heidelberg, Germany; Faculty of Medicine and University Hospital Carl Gustav Carus, TU Dresden, Dresden, Germany. Helmholtz-Zentrum Dresden–Rossendorf (HZDR), Dresden, Germany

**Keywords:** Myeloproliferative disease, Cancer genetics

## Abstract

Several studies have reported that chronic myeloid leukaemia (CML) patients expressing e14a2 *BCR::ABL1* have a faster molecular response to therapy compared to patients expressing e13a2. To explore the reason for this difference we undertook a detailed technical comparison of the commonly used Europe Against Cancer (EAC) *BCR::ABL1* reverse transcriptase quantitative polymerase chain reaction (RT-qPCR) assay in European Treatment and Outcome Study (EUTOS) reference laboratories (*n* = 10). We found the amplification ratio of the e13a2 amplicon was 38% greater than e14a2 (*p* = 0.015), and the amplification efficiency was 2% greater (*P* = 0.17). This subtle difference led to measurable transcript-type dependent variation in estimates of residual disease which could be corrected by (i) taking the qPCR amplification efficiency into account, (ii) using alternative RT-qPCR approaches or (iii) droplet digital PCR (ddPCR), a technique which is relatively insensitive to differences in amplification kinetics. In CML patients, higher levels of *BCR*::*ABL1/GUSB* were identified at diagnosis for patients expressing e13a2 (*n* = 67) compared to e14a2 (*n* = 78) when analysed by RT-qPCR (*P* = 0.0005) but not ddPCR (*P* = 0.5). These data indicate that widely used RT-qPCR assays result in subtly different estimates of disease depending on *BCR::ABL1* transcript type; these differences are small but may need to be considered for optimal patient management.

## Introduction

*BCR::ABL1* is the primary driver of chronic myeloid leukaemia (CML) but this chimeric gene exists in several different isoforms that need to be recognized for optimal patient management [1]. The two most common *BCR::ABL1* mRNA transcripts, both of which encode a 210 kDa BCR::ABL1 protein (p210), are characterized by splicing of *BCR* exon 13 or *BCR* exon 14 to *ABL1* exon 2, and are designated as e13a2 and e14a2, respectively [1–3]. *BCR* exon 14 is 75 bp in size and thus the e14a2 mRNA encodes an additional 25 amino acids compared to e13a2 [1]. Together, these two transcripts are seen in 98% of cases of CML, with e14a2 nearly twice as prevalent as e13a2 and up to 10% of cases expressing both variants [2]. The remaining 2% of CML cases express atypical *BCR::ABL1* fusions involving different *BCR* and/or *ABL1* exons; recognition of these cases is important for their clinical management [4]. The *BCR::ABL1* transcript type expressed by individual patients is determined largely by the precise positions of the genomic breakpoints on chromosomes 22 and 9 [5], and is thus stable over time.

**Fig. 1:**
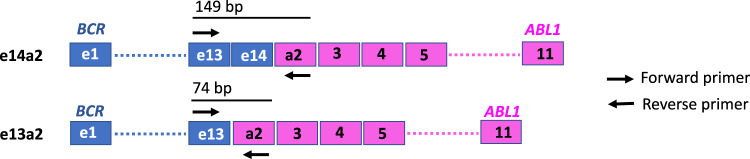
Schematic of e14a2 and e13a2 *BCR::ABL1* with positions of EAC primers.

For routine molecular monitoring of response to treatment, most laboratories use the Europe Against Cancer (EAC) reverse transcriptase quantitative polymerase chain reaction (RT-qPCR) assay, or variants thereof, which use a single primer pair/probe combination to detect and quantify e13a2 and/or e14a2 in the same procedure [6]. Whilst this allows for a single test to be used for the vast majority of CML patients, it presents a potential technical issue as the e14a2 amplicon is approximately twice as large as e13a2 (149 bp vs 74 bp; Fig. 1), and it is known that the qPCR quantification cycle (Cq) generally increases as a function of amplicon size [7]. Indeed, a small study has described a bias towards preferential amplification of e13a2 over e14a2 when using RT-qPCR compared to digital droplet PCR (ddPCR), as well as distinct RT-qPCR amplification profiles for each transcript type [8].

Several clinical studies have indicated that patients expressing e13a2 *BCR::ABL1* have an inferior molecular response to treatment at multiple timepoints compared to those expressing e14a2 [9–11], although this does not appear to translate into a measurable effect on survival [12]. The possibility that the observed difference in response could be explained by variance in RT-qPCR assay performance between the two major transcripts has not yet been fully investigated. As treatment cessation for CML patients who achieve sustained deep molecular response (DMR) to tyrosine kinase inhibitor (TKI) therapy becomes routine practice, it is increasingly important to ensure molecular monitoring is as accurate as possible for all patients, and that treatment decisions are based upon robust laboratory data.

The study described here was designed to investigate the possibility that the observation of higher measurable residual disease (MRD) levels in e13a2 patients could be due to differing efficiencies in PCR amplification between the two transcripts, which is a crucial parameter in RT-qPCR [13, 14].

## Methods

### EUTOS technical study

#### RT-qPCR study design

14 reference laboratories from the European Treatment and Outcome Study (EUTOS) for CML network that routinely use the EAC *BCR::ABL1* assay (Fig. 1) and *ABL1* as a reference gene were sent study materials that were prepared in Salisbury. The materials included (i) three sets of primers and probes: set 1 was specific to e13a2 [15], set 2 used the EAC design for *BCR::ABL1* [6] and set 3 was specific to e14a2 (Supplementary Table 1); [15] (ii) 1 set of plasmid dilutions and 3 sets of cell lysate dilutions for both e13a2 and e14a2. RNA extraction, cDNA synthesis and EAC *BCR::ABL1* qPCR were performed at each site according to local procedures and included the use of laboratory-specific conversion factors (CF) to express results on the International Scale (IS) according to the protocol detailed in Supplementary Methods A and summarised in Supplementary Fig. 1. Results were assessed to ensure the study protocol had been complied with and were excluded from 3 laboratories due to the use of variable RT-qPCR thresholds across different runs. The results from one further laboratory were also excluded as the average A_R_ of the local *BCR::ABL1* assay exceeded 1.5 fold of the interquartile range of all laboratories [16]. The final dataset thus consisted of results from 10 laboratories. Since both the ERM-AD623 certified reference plasmid [17] and WHO International Genetic Reference Panel for the quantitation of *BCR::ABL1* [18] are both based on e14a2 *BCR::ABL1*, results were considered relative to this transcript type.

#### e14a2 and e13a2 plasmids

The ERM-AD623 certified reference plasmid includes the e14a2 *BCR::ABL1* junction sequence as well as parts of the *ABL1*, *BCR* and *GUSB* genes that are used commonly as a reference to control for variation in sample quality and RT-qPCR efficiency. The plasmid is supplied as 6 different concentrations over a range of 10 to 1 × 10^6^ copies/µL and is commonly used as a calibration standard by laboratories performing molecular monitoring for CML [17]. The e13a2 plasmid was identical in construction to ERM-AD623 but contains an e13a2 *BCR::ABL1* fragment in place of e14a2 (Supplementary Fig. 2). A 10-fold dilution series from approximately 10 to 1 × 10^6^ copies/µL was prepared and calibrated to ERM-AD623 reference material using *ABL1* copy number data (Supplementary Fig. 3). Each plasmid has a 1:1 ratio of *BCR::ABL1/ABL1* copy numbers. Laboratories using the EAC assay routinely use an e14a2 plasmid to generate standard curves and use this curve to assign copy numbers to patient samples regardless of the transcript type being expressed, resulting in potential discrepancies in amplicon size between the standard curve and sample.

#### Cell line material

A five-fold dilution series was prepared by diluting *BCR::ABL1* human cell lines expressing e14a2 (K562) or e13a2 (KCL-22) into a *BCR::ABL1* negative cellular background (HL60). Dilutions of each cell line were targeted to contain approximately 10, 2, 0.4, 0.08, or 0.016% *BCR::ABL1/ABL1*, which was confirmed by RT-qPCR prior to distribution. The initial dilution was generated by adding 6 × 10^5^ *BCR::ABL1* expressing cells (K562 or KCL-22) to 6 × 10^7^ HL60 cells, which were then further serially diluted into HL60 cells at a concentration of 1.5 × 10^6^ cells/ml. Cells were lysed in RLT buffer (Qiagen, Hilden, Germany) according to the manufacturer’s instructions to generate final cell lysates samples containing approximately 5 × 10^5^ cells in 600 µL of lysis buffer.

#### Droplet digital PCR

ddPCR was performed using EAC-based *BCR::ABL1* and *ABL1* assays according to locally established procedures [19], or with the commercially available QXDx *BCR::ABL1* %IS kit (BioRad, Hercules, California, USA), according to the manufacturer’s instructions. Both cell line and plasmid material were tested, however as ddPCR experiments can become saturated at very high levels of template copy number, only 4/6 plasmid dilutions were used for ddPCR experiments, spanning a concentration range of approximately 1 × 10^1^ to 1 × 10^4^ copies/µL. The ratio of *BCR::ABL1/ABL1* was calculated from the reported copy number of each target.

#### Dynamics of RT-qPCR

We measured two parameters to assess the performance of e13a2 and e14a2 amplification: (i) the amplification ratio (A_R_) and (ii) amplification efficiency (E) as previously defined [20]. Amplification efficiency-corrected A_R_ values (designated A_RC_) were calculated [21], as well as the expected number of copies of a target amplicon with the observed values of E for e13a2 and e14a2. These calculations are detailed in Supplementary Methods B.

### Patient cohorts

#### Diagnostic CML cohort

A cohort of CML patients at diagnosis were identified (*n* = 152). Patients shown to be co-expressing both e13a2 and e14a2 were excluded (*n* = 7), leaving a total of 145 cases in the final analysis (e13a2, *n* = 67; e14a2, *n* = 78). Patient samples were analysed using RT-qPCR assays for *BCR::ABL1* and *GUSB* [6]. The same samples were also analysed using an in-house RT-ddPCR for *BCR::ABL1* [19]. The *BCR::ABL1* assays used for RT-qPCR and ddPCR both co-amplified e13a2 and e14a2. Results were expressed as %*BCR::ABL1* (RT-qPCR or ddPCR copies) / *GUSB* (RT-qPCR copies). Results were not converted to the International Scale as the %*BCR::ABL1 / GUSB* values greatly exceeded 10%.

#### Subset of patients with sequential monitoring data

Sequential prospective monitoring of MRD at both the mRNA and DNA levels for a subset of 81 CML patients (43 males, 38 females) has been described previously [19]. Finally, data from 67/81 patients with optimized DNA-based assays were used and evaluated (Supplementary methods C). Of these, 27 patients expressed e13a2 and 40 patients expressed e14a2 *BCR::ABL1* transcript type. Monitoring data from these patients were used to determine the time to achieve of a 3-log reduction in disease levels using a measure of individual molecular response (IMR) that is applicable to both RNA and DNA samples, as well as the kinetics of disease reduction [22] as described in detail in Supplementary Methods C.

#### Statistical analysis

Comparisons between groups were performed using the Mann-Whitney U test. Paired comparisons were performed using the Wilcoxon signed-rank test, with Bonferroni correction for multiple tests where appropriate. RT-qPCR and ddPCR measurements were also compared using Bland-Altman analysis [23] with the blandr package for R [24] to assess bias.

## Results

### Impact of *BCR::ABL1* transcript type on amplification performance

**Fig. 2:**
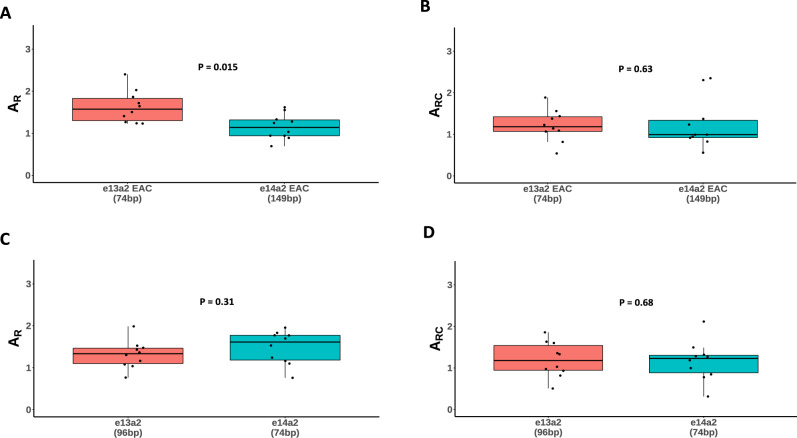
Comparison of e13a2 and e14a2 RT-qPCR *BCR::ABL1/ABL1* amplification ratios (A_R_) for EAC and transcript-specific assays. **A** Without correction for efficiency, the median A_R_ of the shorter EAC e13a2 amplicon was significantly higher than e14a2 (n = 10, median e13a2 ratio = 1.57, e14a2 ratio = 1.14, *P* = 0.015; Mann—Whitney U test). **B** Correcting for amplification efficiency greatly reduces this discrepancy (median e13a2 corrected ratio = 1.18, e14a2 = 0.99, *P* = 0.63). Using transcript-specific assays that are more similar in size, with (**C**) no efficiency correction the shorter e14a2 amplicon has a slightly elevated median A_R_ compared to e13a2, but the difference is not statistically significant (median e13a2 ratio 1.34, e14a2 = 1.61, *P* = 0.31). **D** After correction for efficiency the difference is reduced (median e13a2 corrected ratio = 1.18, e14a2 = 1.23, *P* = 0.68).

To investigate the kinetics of *BCR::ABL1* amplification by RT-qPCR by the widely-used EAC protocol, we undertook a detailed multicentre performance evaluation using control materials according to the schema shown in Supplementary Fig. 1. As the plasmid *BCR::ABL1/ABL1* copy number ratio is 1:1 regardless of plasmid concentration, the median laboratory specific amplification ratio (number of target molecules relative to the number reference molecules; A_R_, see Supplementary Methods B) for each transcript type was determined using all plasmid samples for the routine, EAC-based *BCR::ABL1* assay for each laboratory (runs 5 and 6, Supplementary Fig. 1). The e13a2 A_R_ values were higher than e14a2 in 8/10 laboratories and, overall, the laboratory specific A_R_ values were 38% higher for e13a2 compared to e14a2 (*n* = 10, median e13a2 A_R_ = 1.57 versus e14a2 A_R_ = 1.14, *P* = 0.015, Table 1, Fig. 2A). To determine if the observed difference in A_R_ could be explained by differences in amplification efficiency, we estimated E (Supplementary Methods B, eqn. 2) for the e13a2, e14a2 and *ABL1* assays for each centre using the results from plasmid samples (runs 5 and 6). Overall, amplification of e13a2 was 2% more efficient than e14a2, although this difference did not reach statistical significance (e13a2 median E = 0.972 versus e14a2 = 0.953, *P* = 0.17, Supplementary Fig. 4A). The amplification efficiency-corrected A_RC_ values showed a reduction in the difference between e13a2 and e14a2; the median e13a2 A_RC_ remained slightly greater than e14a2, but the difference was no longer significant (median e13a2 A_RC_ = 1.18 versus e14a2 = 0.99, *P* = 0.63 Fig. 2B). This correction suggests the differences in amplification efficiency explain at least some of the observed difference in A_R_ between e13a2 and e14a2.

**Table 1:** Median (*n* = 10) uncorrected (A_R_) and corrected (A_RC_) amplification ratios and amplification efficiency (E) derived from plasmid material for the EAC and transcript specific assays.

EAC assay	e13a2 (74 bp)	e14a2 (149 bp)	*P*
A_R_ (min, max)	1.57 (1.23, 2.40)	1.14 (0.69, 1.62)	0.015
A_RC_ (min, max)	1.18 (0.54, 1.89)	0.99 (0.56, 2.35)	0.63
E (min, max)	0.972 (0.95, 1.05)	0.953 (0.91, 1.05)	0.17

Transcript type-specific assay	e13a2 (96 bp)	e14a2 (74 bp)	*P*
A_R_ (min, max)	1.34 (0.77, 1.99)	1.61 (0.76, 1.96)	0.31
A_RC_ (min, max)	1.18 (0.51, 1.86)	1.23 (0.31, 2.11)	0.68
E (min, max)	0.962 (0.94, 1.03)	0.982 (0.95, 1.08)	0.069

Amplicon sizes for each assay are indicated in brackets. p-values: Mann-Whitney test, comparing transcript sizes. *p* < 0.05 was considered statistically significant.

To understand in more detail the impact of different amplicon size, the study design included e13a2-specific and e14a2-specific qPCR assays that are used routinely by some centres, particularly in Australasia [15]. The amplicon length for these assays is more comparable between *BCR::ABL1* isoforms; e13a2 (96 bp) and e14a2 (74 bp). In contrast to the EAC assay, we found the A_R_ for the specific assays to be higher for e14a2 in 7/10 laboratories, but the difference overall was not statistically significant (median A_R_ for e13a2 = 1.34 versus e14a2 = 1.61, *P* = 0.31, Fig. 2C, Table 1). Furthermore, we found that the shorter e14a2 amplicon amplified 2% more efficiently than e13a2 using the transcript-specific assays (median E for e13a2 = 0.962 versus e14a2 = 0.982, *P* = 0.069, Supplementary Fig. 4B). Correction for amplification efficiency resulted in a median A_RC_ that was closer to 1 for both transcripts, as well as a reduced difference in A_R_ although the e14a2 ratio remained slightly greater than e13a2 (median e13a2 A_RC_ = 1.18 versus e14a2 A_RC_ = 1.23, *p* = 0.68, Fig. 2D, Table 1). Interestingly, these results are the inverse of those obtained from the EAC qPCR assay, with the e14a2 specific primers outperforming those specific to e13a2. In both cases, however, the more efficient amplification was seen for the shorter amplicon (Table 1).

### Impact of *BCR::ABL1* transcript type on standard curves used for quantification of copy number

**Fig. 3:**
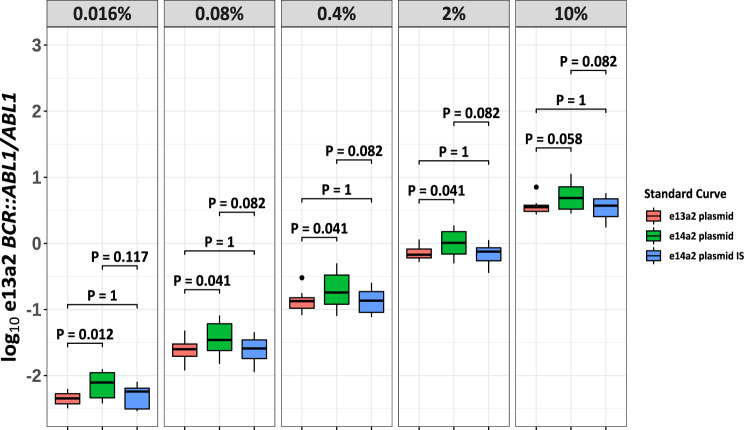
Influence of transcript type used for the standard curve. Log_10_ *BCR::ABL1/ABL1* percentages derived from serially diluted e13a2 *BCR::ABL1* cell line (KCL-22) lysates, calculated using either an e13a2 (red) or e14a2 (green) standard curve, or with the e14a2 standard curve and results converted to the IS (blue). e13a2 *BCR::ABL1* results were consistently higher when calculated with an e14a2 standard curve compared to using an e13a2 standard curve (0.016% dilution, *P* = 0.012; 0.08% dilution, *P* = 0.041; 0.4% dilution, *P* = 0.041; 2% dilution, *P* = 0.041; 10% dilution, *P* = 0.058; Wilcoxon signed-rank test with Bonferroni correction for multiple comparisons). Using results expressed on the IS resolved this difference but with an apparent increase in variability.

To investigate if the *BCR::ABL1/ABL1* qPCR results could be influenced by the transcript type of the standard curve, *BCR::ABL1/ABL1* values for KCL-22 (e13a2 cell line) dilutions were calculated using the local EAC qPCR assay and either the e13a2 or the ERM-AD623 e14a2 plasmid standard curves used to assign copy numbers (runs 5 and 6). If the performance of the assay was similar for both transcript types, then the transcript type of the standard curve should not affect the calculated *BCR::ABL1/ABL1* values. For all dilutions, the results (log_10_ scale) were higher when calculated using the e14a2 standard curve, compared to using the e13a2 standard curve (Fig. 3). This difference was statistically significant at the 0.016%, 0.08%, 0.4 and 2% dilution points and approached significance at the 10% dilution. After applying laboratory-specific CFs (derived from previous EUTOS standardisation rounds) to the results, there was no significant difference between *BCR::ABL1/ABL1* derived from the e13a2 standard curve, and *BCR::ABL1*^IS^, indicating that the use of a CF may go some way to mitigating the difference in efficiency (Fig. 3).

However, estimation of e13a2 using the e14a2 standard curve with or without the CF resulted in increased variability compared to using the e13a2 standard curve (Fig. 3). The mean coefficient of variation (CV) across all dilutions of the e13a2 cell line when using the e13a2 standard curve was 33%, compared to 41% when using the e14a2 standard curve and CF. In contrast, the mean CV of the *BCR::ABL1/ABL1* results from the e14a2 cell line decreased from 43 to 32% when laboratory-specific CFs were applied. This suggests that CFs are not completely optimised for e13a2 *BCR::ABL1*, and interestingly, that e13a2 amplification may be inherently less variable than e14a2, possibly as a result of the much shorter amplicon.

### Effect of using ddPCR

**Fig. 4:**
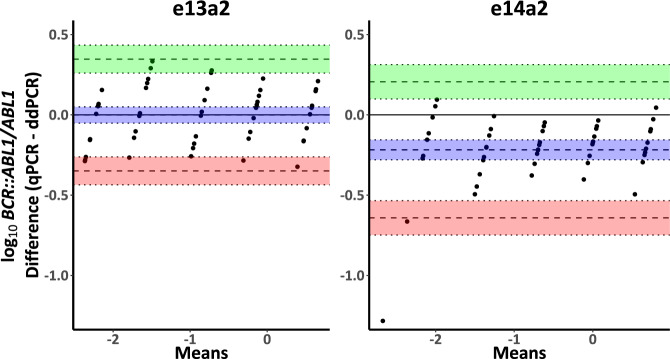
Bland-Altman analysis of the difference between the difference [RT-qPCR (EAC) – ddPCR (QXDx, BioRad)] versus mean BCR::ABL1^IS^. Good concordance for the e13a2 amplicon (mean bias = −0.001, SD = 0.18, 95% CI [0.05, −0.05]) but negative bias for the e14a2 amplicon (mean bias = −0.218, SD = 0.21, 95% CI [−0.28, −0.16]). Each point represents the mean BCR::ABL1^IS^ result of a cell lysate sample from a single laboratory, and the results cluster around the mean results from each dilution point. Blue shading indicates the mean bias (dashed line) and corresponding 95% CI (dotted lines). Green shading indicates the upper LoA and corresponding 95% CI. Red shading indicates the lower LoA and the corresponding 95% CI. SD = standard deviation, CI = confidence interval, LoA = 95% limit of agreement. Log_10_ scale.

To investigate if the differences in performance were specific to RT-qPCR, two laboratories performed ddPCR using their in-house ddPCR protocols, as well as a commercially available, CE marked ddPCR kit (QXDx BCR-ABL %IS, BioRad) for monitoring of *BCR::ABL1* on the IS. Using the EAC primers and plasmid dilutions, there was no difference in ddPCR A_R_ at either laboratory (Salisbury *P* = 0.89; Prague *P* = 0.71, Supplementary Fig. 5). The QXDx assay is not compatible with the *ABL1* moiety in the ERM-AD623 plasmid and thus we were unable to perform the same comparison using the commercial kit, but we were able to compare BCR::ABL1^IS^ RT-qPCR and QXDx IS ddPCR results for the cell line dilutions (*n* = 40). Bland-Altman analysis of the difference between the average log_10_ ddPCR and RT-qPCR results for both transcript types combined showed a bias of −0.11 (SD = 0.22, 95% CI [−0.15,−0.06]), (Supplementary Fig. 6). Individual analysis of each transcript type (Fig. 4) showed a negligible bias of −0.001 for e13a2 however the bias observed for e14a2 was −0.218, suggesting that the EAC RT-qPCR assay does not amplify e14a2 as effectively as the e13a2 transcript when compared to ddPCR. Although we did not observe a difference in A_R_ using EAC ddPCR assays, ddPCR is able to distinguish between the two transcript types, with distinct clusters of droplets defined by *BCR::ABL1* fluorescent amplitude present for each transcript, as has been reported previously [8].

### Impact of transcript type in a patient cohort

**Fig. 5:**
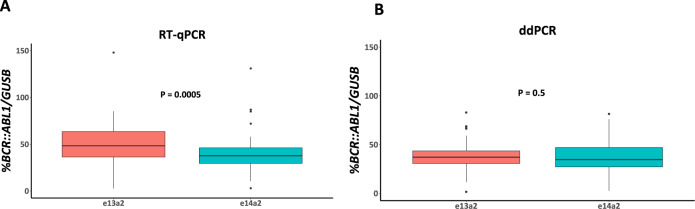
Comparison of %*BCR::ABL1/GUSB* results at diagnosis in patients expressing either e13a2 (*n* = 67) or e14a2 (*n* = 78) *BCR::ABL1*. **A** Using RT-qPCR, the *%BCR::ABL1/GUSB* results were significantly higher in patients expressing e13a2 compared to those expressing e14a2 (median %*BCR::ABL1/GUSB*; e13a2 = 48.3%, e14a2 = 37.7%, *p* = 0.0005). **B** ddPCR measurements for *BCR::ABL1* in the same samples showed no significant difference between transcripts (median %*BCR::ABL1/GUSB* for e13a2 = 37.2% versus e14a2 = 34.6%, *P* = 0.5). Mann—Whitney U test.

**Fig. 6:**
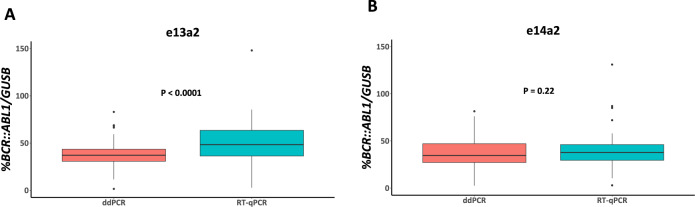
Within-group comparison results for diagnostic samples assessed with RT-qPCR and ddPCR. **A** For the e13a2 patient group, RT-qPCR for *BCR::ABL1* gave significantly higher %*BCR::ABL1/GUSB* results compared to ddPCR for *BCR::ABL1* (e13a2 median ddPCR = 37.16% versus RT-qPCR = 48.32%, *P* < 0.0001, *n* = 67). **B** In the e14a2 group, there was no significant difference in %*BCR::ABL1/GUSB* between methods (median ddPCR = 34.64% versus RT-qPCR = 37.69%, *P* = 0.22, *n* = 78). Wilcoxon signed-rank test.

**Fig. 7:**
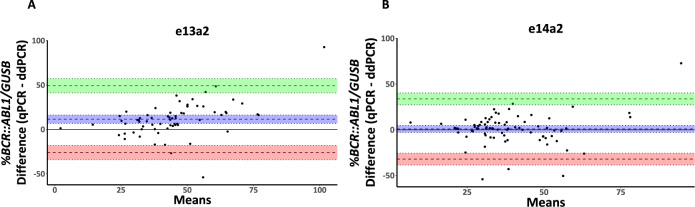
Bland-Altman comparison of RT-qPCR and ddPCR measurement of *BCR::ABL1* in diagnostic samples from patients expressing either e13a2 or e14a2. **A** For e13a2 samples (*n* = 67) the mean bias was 11.52% (95% CI [6.84, 16.21], SD = 19.20). **B** For e14a2 samples (*n* = 78) we found a negligible mean bias of 0.85% (95% CI [–2.94, 4.64], SD = 16.80). Blue shading indicates the mean bias (dashed line) and corresponding 95% CI (dotted lines). Green shading indicates the upper LoA and corresponding 95% CI. Red shading indicates the lower LoA and the corresponding 95%CI. CI confidence Interval, SD standard deviation, LoA 95% limits of agreement.

To assess our findings in CML patients (*n* = 145), we used both RT-qPCR and ddPCR to measure %*BCR::ABL1/GUSB* at diagnosis. Using RT-qPCR, the %*BCR::ABL1/GUSB* was significantly higher for patients expressing e13a2 compared with those expressing e14a2 (e13a2 = 48.3%, e14a2 = 37.7%, *P* = 0.0005, Fig. 5A). Furthermore, the fold difference in median levels for each transcript type was 1.28, close to the theoretical 1.35-fold difference (equation 4 with 30 PCR cycles; Supplementary Methods) that would be expected given the observed 2% difference in amplification efficiency between targets. However, when *BCR::ABL1* was analysed using ddPCR, the difference in %*BCR::ABL1/GUSB* was no longer significant (e13a2 = 37.2%, e14a2 = 34.6%, *P* = 0.5, Fig. 5B). Comparison of %*BCR::ABL1/GUSB* results for each transcript type considered independently showed that the results obtained by RT-qPCR for e13a2 remained significantly greater than those obtained by ddPCR, whereas there was no significant difference for e14a2 (e13a2, *P* < 0.0001; e14a2, *P* = 0.22; Fig. 6). Bland-Altman analysis of RT-qPCR and ddPCR results showed a mean bias for e13a2 of 11.52% (95% CI [6.84, 16.21], Fig. 7A), compared to a mean bias for e14a2 of 0.85% (95% CI [−2.94, 4.638], Fig. 7B). Taken together, these data confirm that e13a2 *BCR::ABL1* is overestimated relative to e14a2 in the RT-qPCR assay at diagnosis, thereby resulting in artificially elevated *BCR::ABL1* results for patients expressing this isoform.

We investigated the effect of *BCR::ABL1* transcript type in a cohort of patients undergoing TKI treatment (*n* = 67). Concordant with the findings of other groups, the time to MMR was shorter for patients expressing e14a2 compared to e13a2, although the difference was not significant in our relatively small series of cases (*P* = 0.077; Supplementary Fig. 7). The analysis of cumulative achievement of a 3 log reduction of *BCR::ABL1* based on IMR measurements (i.e. relative to pretreatment levels for each patient) for both mRNA and DNA assessments showed noticeably less difference between transcript types (Supplementary Fig. 8). Examination of the kinetics of decline using a bi-exponential mixed effect model showed no difference in the α and β slopes between e13a2 and e14a2 for either mRNA or DNA-based assessments (Supplementary Fig. 8).

## Discussion

Molecular monitoring to assess time-dependent therapeutic milestones is an important element in the management of patients with CML [25]. In recent years, several studies have reported that patients expressing e13a2 *BCR::ABL1* have an inferior molecular response at multiple timepoints compared to those expressing e14a2 [9–11]. Although this difference does not affect overall survival [12], it would be expected to have some impact on the achievement of specific milestones as well as eligibility for, or timing of, treatment cessation. Broadly there are two potential, and not necessarily mutually exclusive, explanations for these findings: (i) there is a biological difference between e13a2 and e14a2 BCR::ABL1 that influences response to treatment [26] or (ii) the difference is a technical artefact attributable to the kinetics of RT-qPCR assays employed to measure *BCR::ABL1* mRNA levels. There is some support for the notion that there may be a genuine biological difference between BCR::ABL1 isoforms, for example the finding that transcript type is associated with white cell or platelet counts at presentation [10, 11], progression-free survival [9] or cytogenetic response [27]. However these associations have not been replicated in multiple studies and thus remain tentative. Our study provides evidence that at least part of the difference is technical, and dependent on the assay configuration.

With RT-qPCR, *BCR::ABL1* and reference gene copy numbers are estimated by interpolation of sample Cq to a standard curve derived from calibrated control reagents, and *BCR::ABL1* copies are reported as a percentage of the reference gene, commonly *ABL1, BCR* or *GUSB* [28]. A difference in the efficiency of the target and/or reference gene amplification has the potential to introduce error into the results [29], which is why great care should be taken to ensure amplification is as efficient as possible, and equal for all targets tested [14]. Our results show that the EAC assay performs sub optimally in most laboratories when the target is e14a2, as compared to e13a2. In terms of E, the difference appears slight, but there was a significant difference in A_R_ between e13a3 and e14a2 (Fig. 2A). We were able to correct for this difference by incorporating E into the calculation, indicating that a small difference in E is sufficient to have a measurable impact on the outcome. A likely source for the discrepancy in amplification performance is the difference in amplicon length generated by the EAC assay, although the sequence itself may also be important [6, 7]. Using transcript-specific assays that generate amplicons that are more similar in length, we did not observe a significant difference in A_R_ between the transcript types, but the A_R_ of the shorter amplicon was elevated in comparison to the longer one, supporting the hypothesis that the larger e14a2 EAC amplicon may be impacting amplification performance. Of note, the Adelaide group (which uses transcript-specific assays) did not find any impact of transcript type on the achievement of MMR or MR^4^, although they did find that e14a2 patients were more likely to achieve MR^4.5^ at 48 months [30].

A typical *BCR::ABL1* RT-qPCR test result assumes the equal performance of multiple separate amplifications (*BCR::ABL1* and reference gene for the sample and a 6-point standard curve such as ERM-AD623). This may be a reasonable assumption when comparing like-for-like samples and calibrators, however the commonly used ERM-AD623 plasmid calibrator contains the e14a2 target sequence [17]. As we and others [8] have shown, there is a clear difference in how the EAC-designed *BCR::ABL1* RT-qPCR assay performs depending on the transcript type. It is unsurprising, therefore, that the use of a standard curve containing a different target amplicon may skew the results of an experiment. Indeed, we observed inflated *BCR::ABL1* values from e13a2 expressing cell lines when an e14a2 calibrator was used, compared to results obtained using a matched e13a2 calibrator (Fig. 3). We observed the same pattern of results when an e13a2 calibrator was used to assign *BCR::ABL1* values from e14a2 expressing cell lines (data not shown), which is consistent with an e13a2 standard curve that is amplifying more effectively than the e14a2 standard curve. Although the application of laboratory-specific CFs helps to mitigate against this difference, the increase in variation of the results suggests that CFs may not be fully optimised for the e13a2 transcript. Recent work by Dominy et al [31] also investigated the effect of transcript-specific standard curves, and our results corroborate and extend their findings. All currently available reference materials for *BCR::ABL1* are based on e14a2, which likely accounts for the relative lack of assay optimisation for e13a2. In theory these issues could be addressed by production of e13a2-based reference materials that would enable assay optimisation (and potentially new assay design), estimation of E and correction of results. The ‘Pfaffl method’, for example, is frequently used in relative quantitation experiments [32] and has been proposed for use in absolute quantitation [29, 33]. However development of certified reference materials is a lengthy and complex process; furthermore it is not entirely clear how to deal with patients who express both e13a2 and e14a2.

An alternative approach is to use ddPCR, a technique which is relatively insensitive to differences in amplification efficiency as well as having other advantages such as producing results that are less variable that those produced by RT-qPCR and the lack of requirement for a standard curve [34–37]. Our initial data using control materials indicated that ddPCR results do not show the transcript-related differences that were seen using RT-qPCR. This was confirmed in CML patients, for whom we found *BCR::ABL1/GUSB* levels at diagnosis were apparently elevated in e13a2 cases compared to e14a2 when using RT-qPCR, but no difference was seen with ddPCR. The negative bias in RT-qPCR e14a2 amplification when compared with ddPCR is consistent with reduced efficiency of the e14a2 EAC assay as the source of experimental error. It is important to note that variations in laboratory protocols, including the use of different reference genes, are likely to lead to variable levels of bias (if any) between transcript types in different laboratories, and testing centres with concerns should undertake their own internal investigations to determine the performance of their assays for both e13a2 and e14a2 *BCR::ABL1*.

When RT-qPCR results were normalized to pre-treatment levels, there was no difference between transcript types with respect to achievement of a 3-log reduction in levels of disease, and no difference in the α or β slopes was apparent (Supplementary Fig. 8). Although this approach is helpful to evaluate prognostically significant differences in the rate of disease reduction during the first weeks of therapy [38, 39], and is the only approach to monitor molecular response for cases with rare, atypical *BCR::ABL1* variants [4], it is of limited value for most patients because the results cannot be related to the IS. Similarly, DNA-based results can provide useful information in patients in DMR [19, 40] but this technically difficult approach appears to add little value for routine monitoring.

In conclusion, there is a growing body of evidence that points to discrepancies in the performance of the EAC RT-qPCR assay in relation to *BCR::ABL1* transcript type. This issue is almost certainly not limited to the EAC primer/probe set, but likely affects other assays with similar differences in amplicon sizes between e13a2 and e14a2. It is important to emphasize, however, that the discrepancy is subtle and, although its consequences are apparently detectable in some large studies [9–11], the effect on individual cases is expected to be very small [31]. Nevertheless, we recommend caution in making clinical decisions based on patient transcript type and stress the need to consider trends in sequential MRD results in addition to the achievement of defined milestones at specific timepoints.

## Supplementary information

Supplemental Material

## Data Availability

The datasets generated during the study are available from the corresponding author on reasonable request.
