# Supplementary material for: Impact of BCR::ABL1 transcript type on RT-qPCR amplification performance and molecular response to therapy
Source: Leukemia. 2022 Jun 8;36(7):1879–86. doi: 10.1038/s41375-022-01612-2 (PMC9252903; doi:10.1038/s41375-022-01612-2)
Supplement: Supplementary file 1 — Supplemental Material [file 41375_2022_1612_MOESM1_ESM.pdf]

SUPPLEMENTARY INFORMATION

Impact of *BCR::ABL1* transcript type on RT-qPCR amplification performance and molecular response to therapy

Matthew Salmon, Helen E. White, Hana Zizkova et al.

SUPPLEMENTARY METHODS

A. EUTOS Technical study full protocol (provided to participating laboratories)

Participating laboratories were blinded to plasmid and cell line transcript type which were as follows: Plasmid X = e13a2, Plasmid Y = e14a2 ERM-AD623 (ref. 1), cell lysates A-E = e13a2 (KCL22), cell lysates F-J = e14a2 (HL60). RT-qPCR primer and probe sets were as follows: Set 1 = e13a2 specific *BCR::ABL1*, Set 2 = EAC *ABL1*, Set 3 = e14a2 specific *BCR::ABL1* (Supplementary table 1).

Material provided:

i. 3 sets of qPCR primers/probes targeting *ABL1* or *BCR::ABL1*. All probes are dual-labelled with 5'FAM/ 3'BHQ1
ii. 2 sets of serially diluted plasmid samples labelled Plasmid X (1-6) and Plasmid Y (1-6).
iii. Set of cell lysate samples (labelled A-J, in triplicate) in RLT or Trizol as requested.
iv. Results spreadsheet.

Study Overview:

Samples: Each plasmid sample and cell line lysate will be assessed by qPCR using all 3 primer/probe sets, plus your usual *ABL1* and *BCR::ABL1* primer/probe sets (4 primer/probe sets in total). Each qPCR run will detect **either** *ABL1* **or** *BCR::ABL1*.

qPCR: Each sample must be assessed by qPCR using the appropriate primer/probe sets (see Supplementary Figure 2). Please ensure you use the same number of replicates across all runs. In total, you will need to perform 6 qPCR runs:

- Run 1: test all plasmid and cDNA samples (n=23) with primer/probe set 1
- Run 2: test all plasmid and cDNA samples (n=23) with primer/probe set 2
- Run 3: test all plasmid and cDNA samples (n=23) with primer/probe set 3
- Run 4: test all plasmid and cDNA samples (n=23) with primer/probe set 2
- Run 5: test all plasmid and cDNA samples (n=23) with your local *BCR::ABL1* primer/probe set
- Run 6: test all plasmid and cDNA samples (n=23) with your local *ABL1* primer/probe set

RNA Processing: RNA should be extracted and sufficient cDNA synthesised from the cell lysate samples following your standard protocol. One lysate should be used per two qPCR runs (see "Overview" tab of results spreadsheet and supplementary figure 2).

Standard Curves: For **runs 1 and 2**, the **plasmid X** dilution series should be assigned as the standard curve. For **runs 3-6**, the **plasmid Y** dilution series should be assigned as the standard curve. Table 1 shows the transcript copies/µL for each tube. Please ensure you assign your standards the appropriate copy number based on the amount of plasmid used in each run. (e.g. for 2µL: Plasmid 1 = 40 copies, Plasmid 2 = 400 copies…)

Sample	Transcript copies/µL
Plasmid 1	20
Plasmid 2	200
Plasmid 3	2,000
Plasmid 4	20,000
Plasmid 5	200,000
Plasmid 6	2,000,000

Table A: Standard curve transcript copy numbers

Standard qPCR conditions:

Reagent	Vol (µL, n=1)	Final concentration
10µM F primer	2	1µM
10µM R primer	2	1µM
5µM Probe	0.5	0.125µM
RQ-PCR Master Mix*	x	x
cDNA**	2-5µL	-
Water	To 20µL	-

Table B: qPCR components.

Number of Cycles	Temp (°C)	Time
1x	50	2 minutes
1x	95	10 minutes
50x	95	15 seconds
	60	1 minute

Table C: qPCR cycling conditions

<u>Protocol: (see Supplementary figure 2)</u>

Run 1

1) Extract RNA from the first set of cell lysate samples A-J following your standard procedure.
2) Synthesise cDNA from each RNA sample for use in Runs 1 and 2 following your standard procedure. Take an aliquot of each sample to use in Run 1. Store remaining cDNA at -20°C. Store any remaining RNA at -80°C.
3) Using **primer/probe set 1**, perform qPCR Run 1 on the 23 samples (Plasmid X 1-6, Plasmid Y 1-6, cell lysates A-J, No template control) according to the conditions specified in Tables 2 and 3 above.
4) Assign **Plasmid X** as the standard curve for this run, using the copy numbers given in Table 1.
5) Record the results in the "Run 1" tab of the results sheet.

Run 2

1) With the remaining cDNA from Run 1, and using **primer/probe set 2**, perform qPCR Run 2 on 23 samples (Plasmid X 1-6, Plasmid Y 1-6, cell lysates A-J, No template control) according to the conditions specified in Tables 2 and 3 above.
2) Assign **Plasmid X** as the standard curve for this run, using the copy numbers given in Table 1.
3) Record the results in the “Run 2” tab of the results sheet.

Run 3

1) Extract RNA from the second set of cell lysate samples A-J following your standard procedure.
2) Synthesise cDNA from each RNA sample for use in Runs 3 and 4 following your standard procedure. Take an aliquot of each sample to use in Run 3. Store remaining cDNA at -20°C. Store any remaining RNA at -80°C.
3) Using **primer/probe set 3**, perform RQ-PCR Run 1 on the 23 samples (Plasmid X 1-6-, Plasmid Y 1-6, cell lysates A-J, No template control) according to the conditions specified in Tables 2 and 3 above.
4) Assign **Plasmid Y** as the standard curve for this run, using the copy numbers given in Table 1.
5) Record the results in the “Run 3” tab of the results sheet.

Run 4

1) With the remaining cDNA from Run 3, and using **primer/probe set 2**, perform qPCR Run 2 on 23 samples (Plasmid X 1-6-, Plasmid Y 1-6, cell lysates A-J, No template control) according to the conditions specified in Tables 2 and 3 above.
2) Assign **Plasmid Y** as the standard curve for this run, using the copy numbers given in Table 1.
3) Record the results in the “Run 4” tab of the results sheet.

Run 5

1) Extract RNA from the third set of cell lysate samples A-J following your standard procedure.
2) Synthesise cDNA from each extracted RNA sample for use in Runs 5 and 6 following your standard procedure. Take an aliquot of each sample to use in Run 5. Store remaining cDNA at -20°C. Store any remaining RNA at -80°C.

3) Using **your usual *BCR::ABL1* primer/probes**, set up and perform qPCR Run 5 **according to your standard conditions**. Test all 23 samples (Plasmid X 1-6, Plasmid Y 1-6, cell lysates A-J, No template control)
4) Assign **Plasmid Y** as the standard curve for this run, using the copy number given in Table 1.
5) Record the results in the "Run 5" tab of the results sheet.

Run 6

1) With the remaining cDNA from Run 3, and using **your usual *ABL1* primer/probes**, set up and perform qPCR Run 6 according **your standard conditions**. Test all 23 samples (Plasmid X 1-6, Plasmid Y 1-6, cell lysates A-J, No template control)
2) Assign **Plasmid Y** as the standard curve for this run, using the copy numbers given in Table 1.
3) Record the results in the "Run 6" tab of the results sheet.

B. Dynamics of RT-qPCR

We used two parameters to assess the performance of e13a2 and e14a2 amplification; 1) amplification ratio (A_R) and 2) amplification efficiency (E). A_R can be defined as the number of target molecules relative to the number of some reference molecule at the Cq of an RT-qPCR experiment, as calculated by equation 1. (2)

$$Amplification\ Ratio = 2^{(Cq_r - Cq_t)} \qquad [1]$$

Where Cq_r is the quantification cycle of the reference molecule (*ABL1*), and Cq_t is the quantification cycle of the target molecule (*BCR::ABL1*). The A_R presented here therefore represents the ratio of *BCR::ABL1*/*ABL1* copy numbers.

The E of RT-qPCR describes the increase in copies of the target molecule from cycle to cycle. A reaction is 100% efficient when there is a perfect doubling of target molecules between each PCR cycle. In equation 1, E of the reference and target assays is assumed to be 100%. This is a common assumption, but E is rarely perfect in real-world experiments, and factors such as primer design, template secondary structures, and reaction chemistry can reduce efficiency and render this assumption invalid. (3) It is also possible for the calculated efficiency to exceed 100% (i.e., the number of target molecules more than doubles with each cycle of PCR), a phenomenon that is usually indicative of PCR inhibition at higher template concentrations in the standard curve. To

account for less than perfect amplification, E can be estimated experimentally using equation 2 (ref. (2).

$$E = 10^{\left(\frac{-1}{m}\right)} - 1 \quad [2]$$

Where m is the gradient calculated from the linear regression of Cq against $\log_{10}$ template concentration for a series of samples of known concentration (i.e. a standard curve). For a 100% efficient reaction, E = 1, which corresponds to a standard curve gradient, m, of -3.33 for a 10-fold dilution series. (2) As the absolute initial ratio of *BCR::ABL1/ABL1* is known to be exactly 1:1 in each plasmid used in this study, the A_R as determined by a theoretical RT-qPCR in which all targets are amplified with equal efficiency should be 1. In other words, the Cq of *ABL1* and *BCR::ABL1* would be identical. Additionally, if there is no difference in E between e13a2 and e14a2, there should also be no difference in A_R between the two transcript types. Amplification efficiency-corrected A_R values (designated A_{RC}) were calculated as per Equation 3. (4)

$$Corrected\ Amplification\ Ratio = \frac{(1 + E_r)^{Cq_r}}{(1 + E_t)^{Cq_t}} \quad [3]$$

Where E_r and E_t represent the amplification efficiency of *ABL1* and *BCR::ABL1*, respectively. The number of copies of a target amplicon, N, that will be present after x reaction cycles is described by Equation 4

$$N_x = N_0(1 + E)^{(x-1)} \quad [4]$$

Where N_0 is the initial number of template copies and E is the reaction efficiency between 0 and 1.

C. Analysis of CML patients with molecular follow up data

Sequential prospective monitoring of MRD at both the mRNA and DNA levels for a subset of 81 CML patients (43 males, 38 females) since diagnosis has been described previously. (5) Briefly, 65 patients were treated with imatinib, 14 with nilotinib and 2 with interferon-α (IFN) plus nilotinib as first-line treatment for a median of 28.7 months (range 0.2-45.3). Monthly monitoring by EAC RT-qPCR and DNA qPCR as described below was performed for the first 6 months after TKI start followed by 3 monthly intervals. Therapy was changed in 17 patients due to intolerance or failure after a median

of 6.8 months on 1st line therapy (range 0.2-38.5 months). Non-CML related deaths occurred in 4 patients.

Patient-specific genomic DNA *BCR::ABL1* (g*BCR::ABL1*) qPCR assays were optimized in 71/81 patients as described. (5) Of these 71 patients, 4 were excluded from this study due to a rapid TKI change after the start of first-line TKI treatment (1 patient), combination therapy with interferon-α (2 patients) or higher than normal TKI dose (1 patient). Altogether, data from 67 patients were evaluated. Of these, 27 patients expressed e13a2 and 40 patients expressed e14a2 *BCR::ABL1* transcript type.

g*BCR::ABL1* levels were analysed using patient-specific qPCR with albumin (*ALB*) as the reference gene to normalise results. (5) Individual Molecular Responses (IMR) were calculated relative to the diagnostic sample ($gBCR::ABL1_{RelDg}$) as follows:

$\% \ gBCR::ABL1_{RelDg} = (\% \ gBCR::ABL1_{sample})/(\% \ gBCR::ABL1_{Dg})*100$

Standardized real-time qPCR for *BCR::ABL1* transcript quantification was performed using *GUSB* as reference gene. (6) Similarly, IMRs at the mRNA level were calculated relative to the diagnostic sample ($BCR::ABL1_{RelDg}$) or sample at TKI start ($BCR::ABL1_{RelTKI}$) using formulas:

$\% \ BCR::ABL1_{RelDg} = (\% \ BCR::ABL1_{sample})/(\% \ BCR::ABL1_{Dg})*100$

Samples that passed previously defined quality criteria were considered as evaluable. (21) Minimal quality criteria for measured RNA sample was at least 24,000 copies of control gene *GUSB* ensuring sensitivity of mRNA *BCR::ABL1* at the level of MR^4. (7) For DNA measurements the minimal acceptable number of *ALB* was ≥20,000 copies reflecting 10,000 cells, thus the sensitivity 10^{-4}.

Rather than achievement of major molecular response (MMR; ($BCR::ABL1^{IS}$ ≤0.1%), we used an alternative measure that is applicable to both RNA and DNA samples. Specifically, we investigated the achievement of a 1000-fold (log_{10} = 3) reduction of mRNA or g*BCR::ABL1* levels compared to diagnosis or TKI start. We applied time to event analysis using cumulative incidence estimates (cumulative events) to compare the patients groups presenting with different transcript types and compared cumulative incidence curves using the log-rank test.

A bi-exponential mixed effect model was used to analyse differences in the typical biphasic response patterns measured in terms of *BCR::ABL1* levels. The response is characterized by an initial steep decline (α slope) followed by a second moderate decline (β slope). (8) The slopes and the intercept B were estimated for every single patient, while the intercept A was estimated jointly for all patients (Supplementary Figure 3). The transcript types (e13a2 vs e14a2) were considered as covariates. Wald tests were applied to assess the statistical significance of the group-related fixed-effects.

SUPPLEMENTARY FIGURES

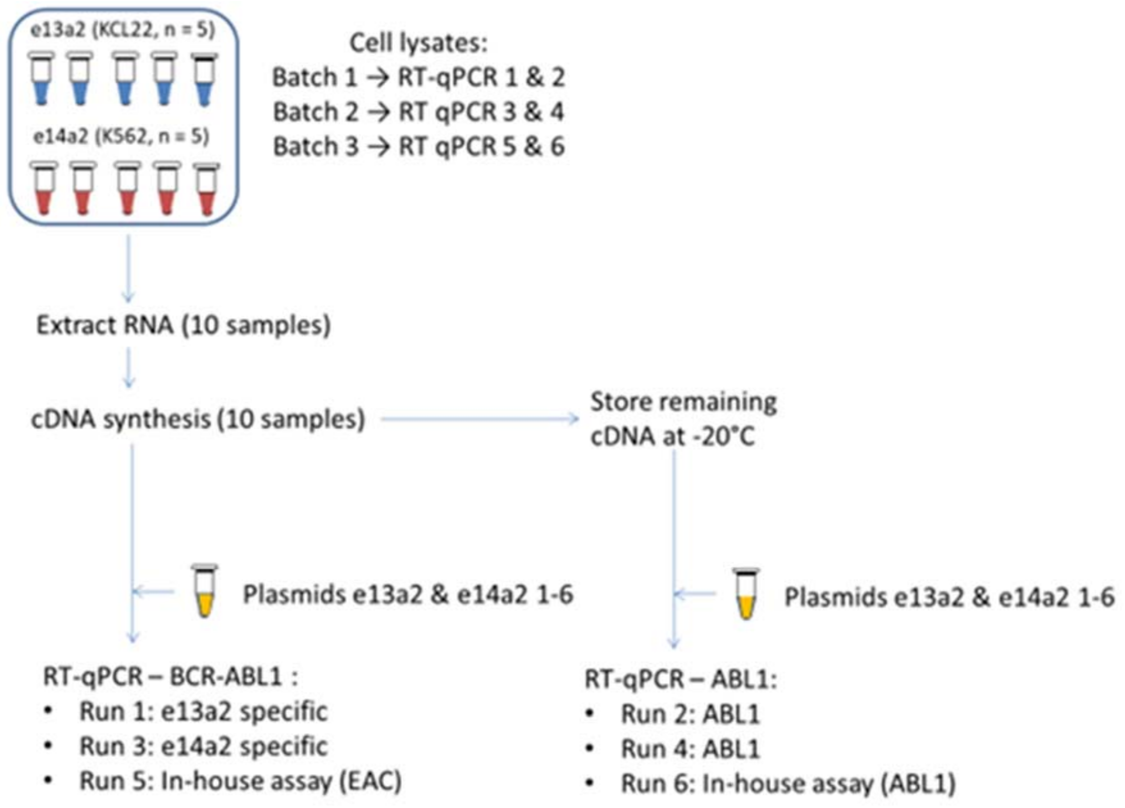

Supplementary Figure 1

Summary of the technical protocol used by EUTOS reference laboratories (n=14)

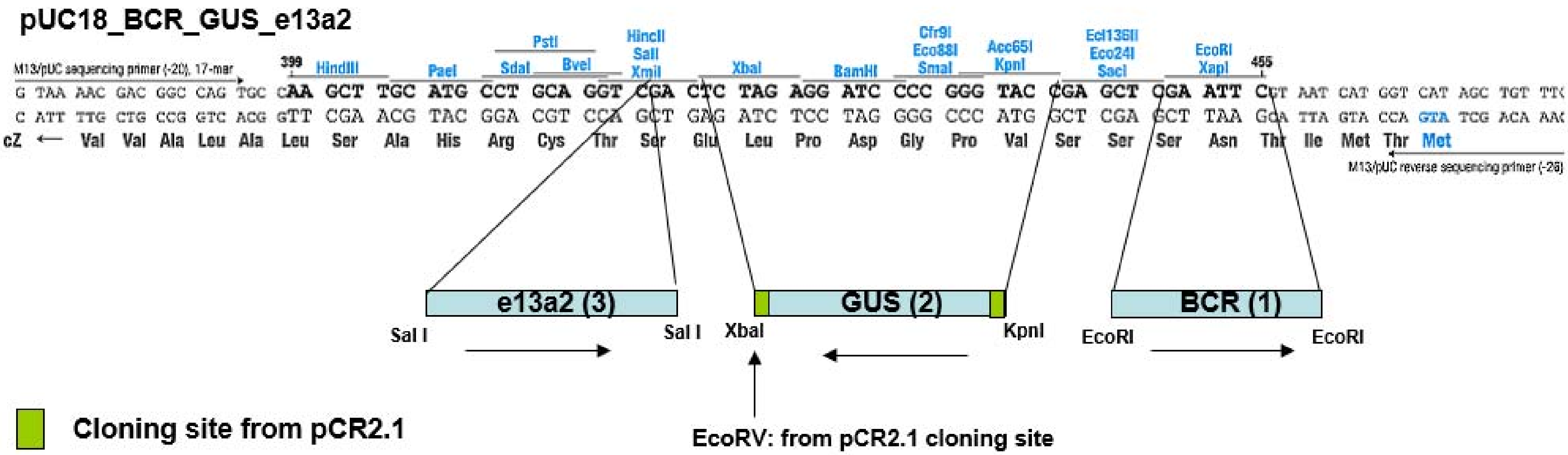


```
e13a2 insert (1324bp): Amplified from e13a2 positive cDNA
CCTCTGCACCAAGCTCAAGAAGCAGAGCGGAGGCAAAACGCAGCAGTATGACTGCAAATGGTACATTCCGCTCACGGATCTCAGCTTCCAGATGGTGGATGAACTGGAGGCAGTGCCCAACATCCCCCTGGTGCCC
GATGAGGAGCTGGACGCTTTGAAGATCAAGATCTCCCAGATCAAGAATGACATCCAGAGAGAGAAGAGGGCGAACAAGGGCAGCAAGGCTACGGAGAGGCTGAAGAAGAAGCTGTCGGAGCAGGAGTCACTGCTGC
TGCTTATGTCTCCCAGCATGGCCTTCAGGGTGCACAGCCGCAACGGCAAGAGTTACACGTTCCTGATCTCCTCTGACTATGAGCGTGCAGAGTGGAGGGAGAACATCCGGGAGCAGCAGAAGAAGTGTTTCAGAAG
CTTCTCCCTGACATCCGTGGAGCTGCAGATGCTGACCAACTCGTGTGTGAAACTCCAGACTGTCCACAGCATTCCGCTGACCATCAATAAGGAAGaagcccttcagcggccagtagcatctgactttgagcctcag
ggtctgagtgaagccgctcgttggaactccaaggaaaaccttctcgctggacccagtgaaaatgaccccaaccttttcgttgcactgtatgattttgtggccagtggagataacactctaagcataactaaaggtg
aaaagctccgggtcttaggctataatcacaatggggaatggtgtgaagcccaaaccaaaaatggccaaggctgggtcccaagcaactacatcacgccagtcaacagtctggagaaacactcctggtaccatgggcc
tgtgtcccgcaatgccgctgagtatctgctgagcagcgggatcaatggcagcttcttggtgcgtgagagtgagagcagtcctggccagaggtccatctcgctgagatacgaagggagggtgtaccattacaggatc
aacactgcttctgatggcaagctctacgtctcctccgagagccgcttcaacaccctggccgagttggttcatcatcattcaacggtggccgacgggctcatcaccacgctccattatccagccccaaagcgcaaca
agcccactgtctatggtgtgtcccccaactacgacaagtgggagatggaacgcacggacatcaccatgaagcacaagctgggcgggggccagtacggggaggtgtacgagggcgtgtggaagaaatacagcctgac
ggtggccgtgaagaccttgaaggaggacaccatggaggtggaagagttcttgaaagaagctgcagtcatgaaagagatcaaacaccctaacctggtgcag
```

Supplementary Figure 2.

E13a2 plasmid map. *BCR* and *GUSB* inserts are described in (9)

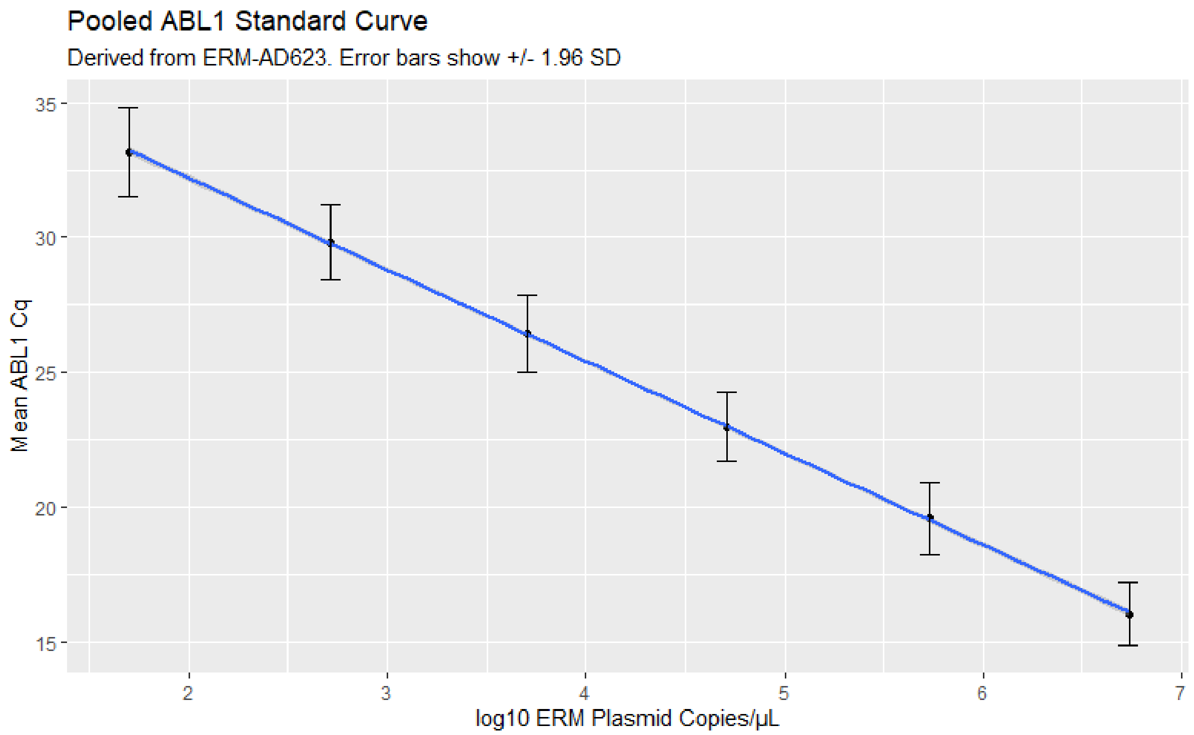

Supplementary Figure 3

Calibration of the e13a2 plasmid. An e14a2 standard curve was constructed using the pooled Cq values from 9 participating EUTOS reference laboratories for *ABL1* amplification from the ERM plasmid (as determined by the laboratory's routine assay) and the certified concentration of that ERM plasmid. The pooled standard curve had an intercept of 39.00, gradient of -3.40 and concentration was significantly correlated with mean *ABL1* Cq ($r^2 = 0.9999$, $p < 0.0001$). The Cq's of *ABL1* amplification of the e13a2 plasmid were pooled from the same 9 laboratories and the concentration of each dilution was determined by interpolation to the pooled e14a2 standard curve.

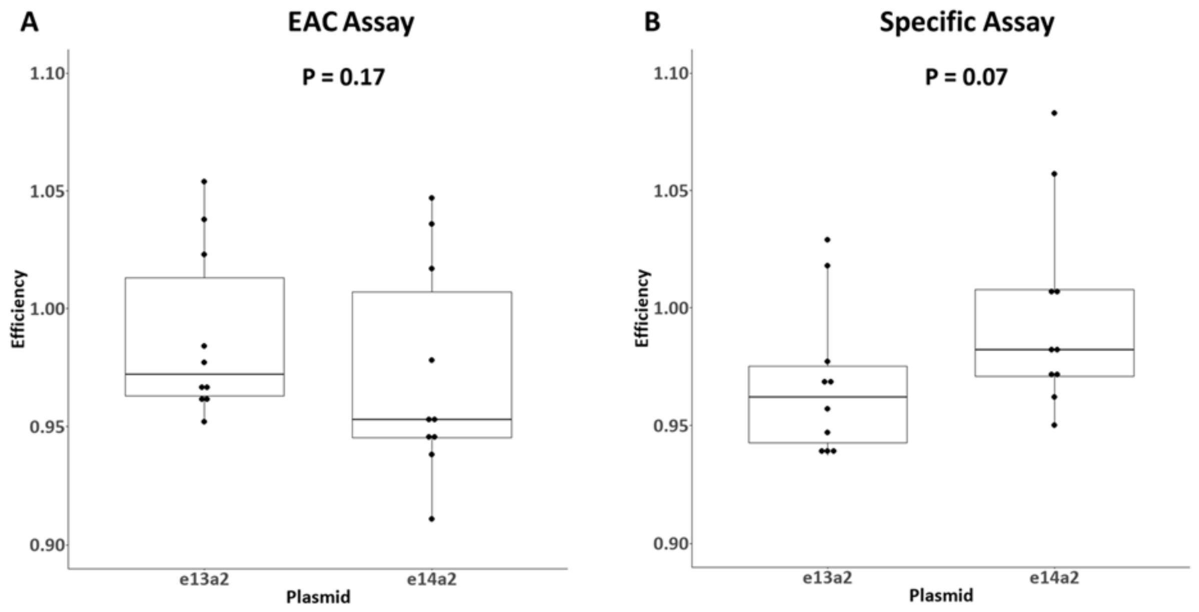

Supplementary Figure 4

Efficiency of amplification for e13a2 and e14a2 *BCR::ABL1* using A) EAC (e13a2 amplicon size = 74bp, e14a2 = 149bp) and B) transcript specific assays (e13a2 amplicon size = 96bp, e14a2 = 74 bp). Comparisons were performed using the Mann-Whitney U test.

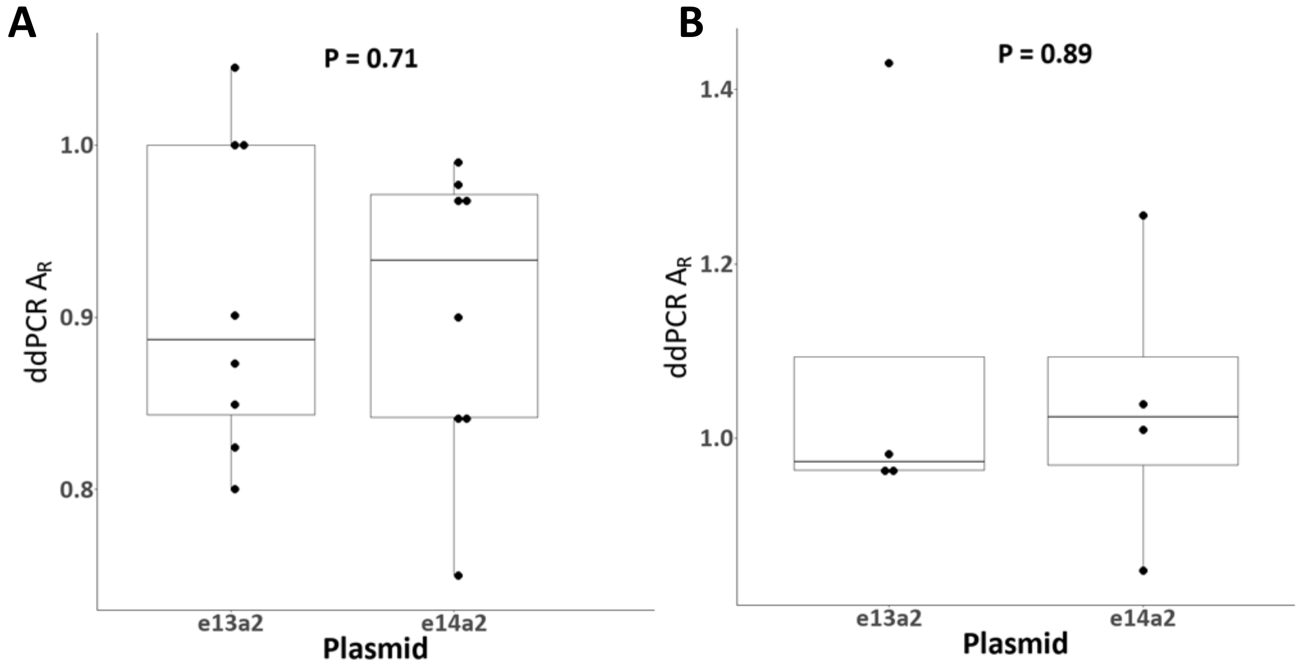

Supplementary Figure 5

Effect of ddPCR. No difference in the ratio of *BCR::ABL1/ABL1* was seen between e13a2 and e14a2 by ddPCR using EAC primers on the 4 lowest levels of plasmid dilutions in (A) Prague and (B) Salisbury. Prague: results from duplicate reactions at each level (n = 8 replicates each for *BCR::ABL1* and *ABL1*, P = 0.71, Mann-Whitney U test). Salisbury: results of triplicate reactions at each level (n = 12 replicates each for *BCR::ABL1* and *ABL1*, p = 0.89).

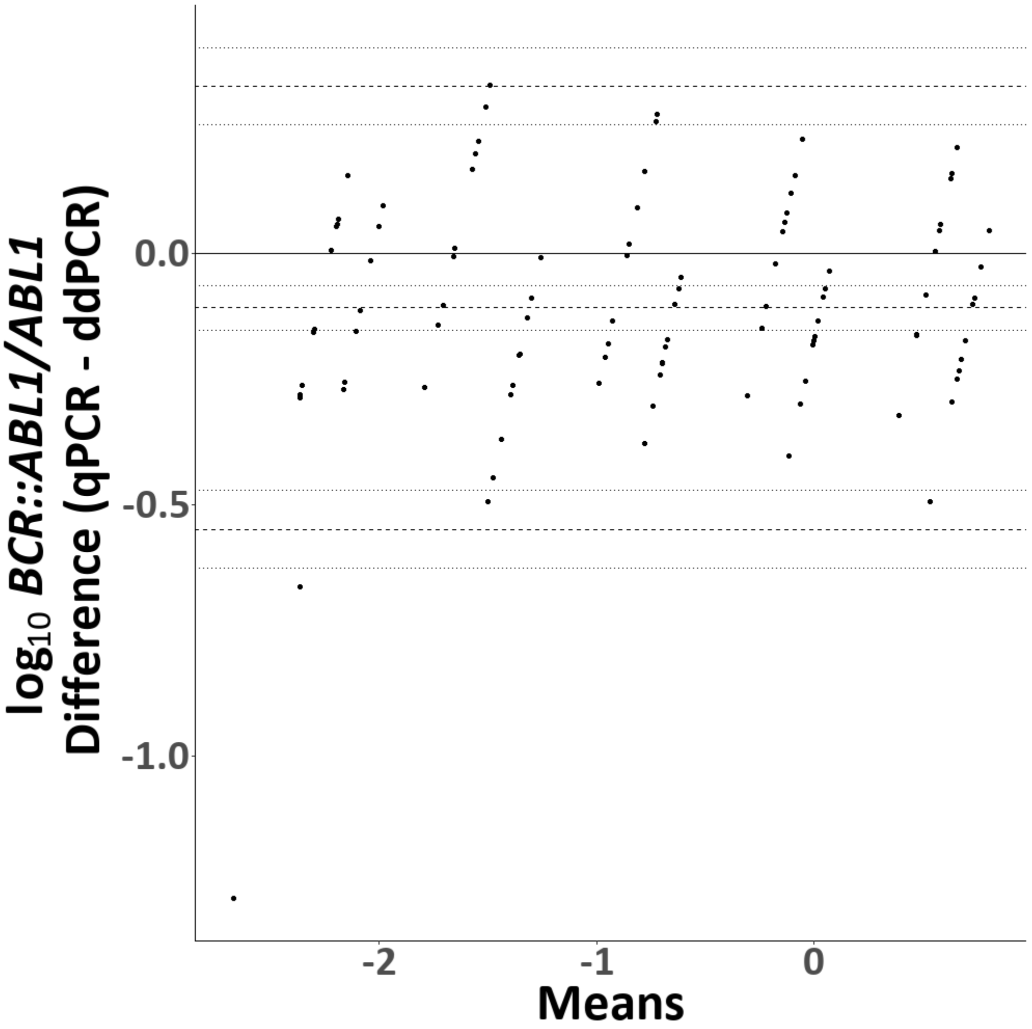

Supplementary Figure 6

Bland-Altman comparison of combined e13a2 and e14a2 results showing a bias of -0.11 (SD = 0.22, 95% CI [-0.15,-0.06]) between the average $\log_{10}$ ddPCR and RT-qPCR results for both transcript types combined.

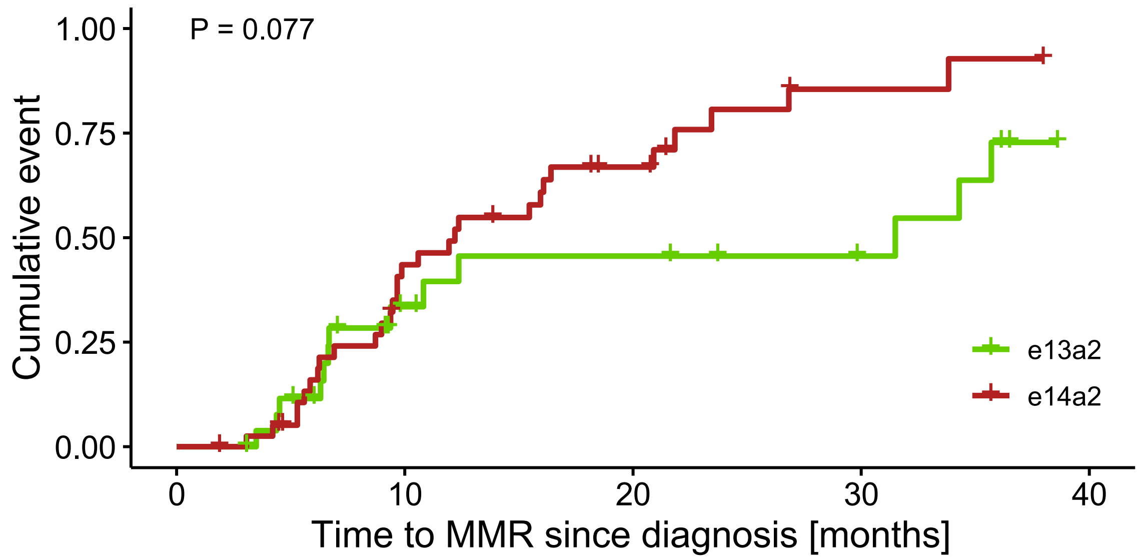

Supplementary Figure 7

Response according to *BCR::ABL1* transcript type. Time from diagnosis to MMR (BCR::ABL1^{IS} ≤0.1%) for e13a2 (n=27) and e14a2 (n=40) patients. Cumulative incidence curves were compared using the log-rank test.

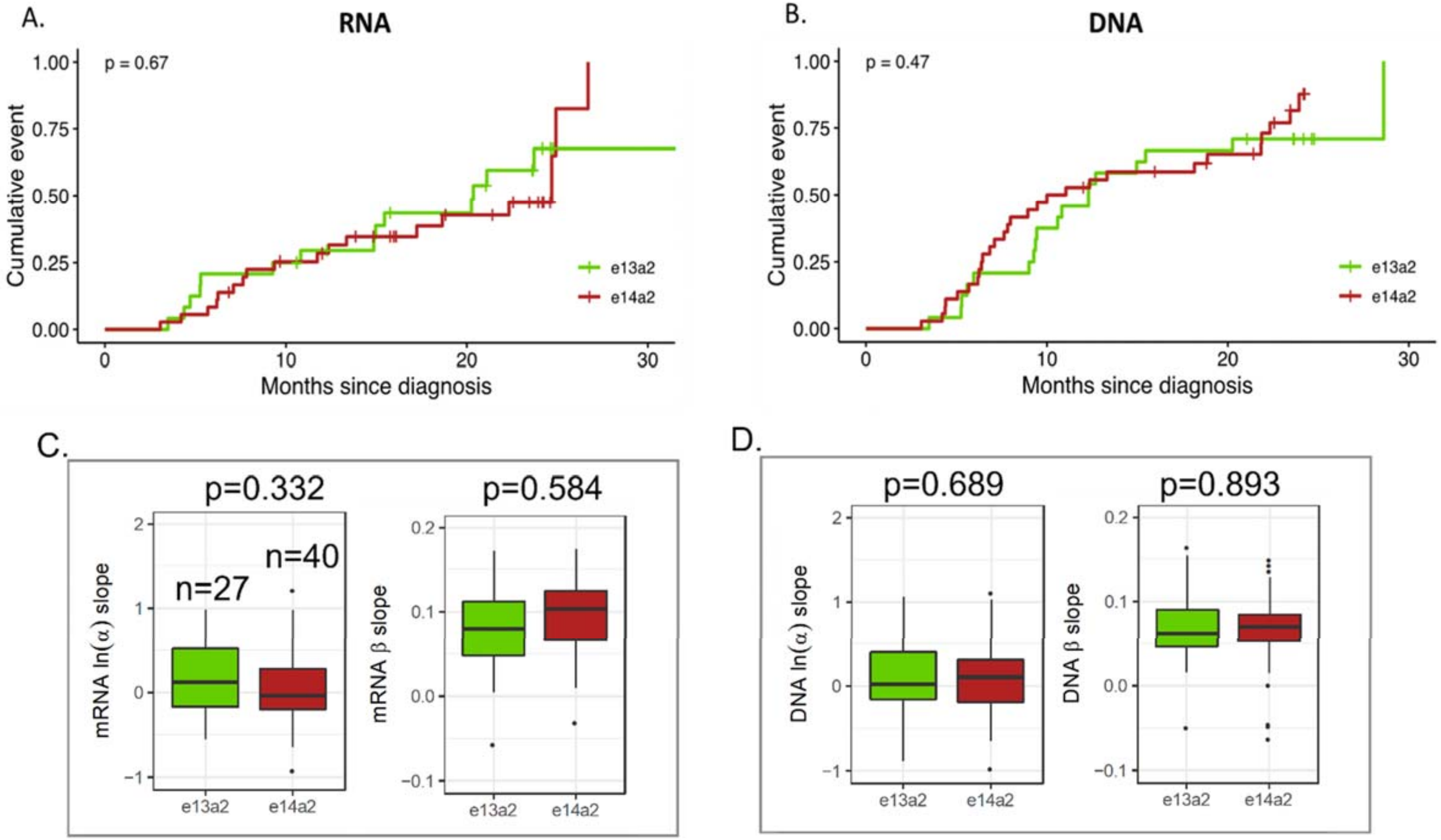

Supplementary Figure 8

Individual molecular responses (time to 3 log reduction) according to *BCR::ABL1* transcript type. (A) Time to 3 log reduction of *BCR::ABL1* mRNA since diagnosis (*BCR::ABL1*$_{RelDg}$); (B) time to 3 log reduction of *BCR::ABL1* genomic DNA since diagnosis (g*BCR::ABL1*$_{RelDg}$). (C) Comparison of α and β slopes from diagnosis according to transcript type using the bi-exponential mixed effects model for mRNA and (D) genomic DNA. Wald tests were applied to assess the statistical significance of the group-related fixed-effects. Modelling was performed using Monlix 2018R2 (Lixoft, Paris, France) and visualised using R. (10)

Name	Sequence (5'-3')
BCR::ABL1 F EAC (ENF501)	TCCGCTGACCATCAAYAAGGA
BCR::ABL1 R EAC (ENF561)	CACTCAGACCCTGAGGCTCAA
BCR::ABL1 Pr EAC (ENF541)	[FAM]CCCTTCAGCGGCCAGTAGCATCTGA[BHQ-1]
ABL1 F EAC (ENF1003)	TGGAGATAACACTCTAAGCATAACTAAAGGT
ABL1 R EAC (ENR1063)	GATGTAGTTGCTTGGGACCCA
ABL1 Pr EAC (ENPr1043)	[FAM]CCATTTTTGGTTTGGGCTTCACACCATT[BHQ-1]
e13a2 BCR::ABL1 F	ATCCGTGGAGCTGCAGATG
e13a2 BCR::ABL1 R	CGCTGAAGGGCTTCTTCCTT
e13a2 BCR::ABL1 Pr	[FAM]CCAACTCGTGTGTGAAACTCCAGACTGTCC[BHQ-1]
e14a2 BCR::ABL1 F	GGGCTCTATGGGTTTCTGAATG
e14a2 BCR::ABL1 R	CGCTGAAGGGCTTTTGAACT
e14a2 BCR::ABL1 Pr	[FAM]CATCGTCCACTCAGCCACTGGATTTAAGC[BHQ-1]

Supplementary Table 1

Primer and probe sequences

Supplementary References

1. White H, Deprez L, Corbisier P, Hall V, Lin F, Mazoua S, et al. A certified plasmid reference material for the standardisation of BCR–ABL1 mRNA quantification by real-time quantitative PCR. Leukemia. 2015;29(2):369-76.
2. Kubista M, Andrade JM, Bengtsson M, Forootan A, Jonák J, Lind K, et al. The real-time polymerase chain reaction. Molecular Aspects of Medicine. 2006;27(2):95-125.
3. Bustin S, Huggett J. qPCR primer design revisited. Biomolecular Detection and Quantification. 2017;14:19-28.
4. Sta A, Kubista M. Quantitative Real-Time PCR Method for Detection of B-Lymphocyte Monoclonality by Comparison of [illegible] and [illegible] Immunoglobulin Light Chain Expression. Clinical Chemistry. 2003(1):9.
5. Machova Polakova K, Zizkova H, Zuna J, Motlova E, Hovorkova L, Gottschalk A, et al. Analysis of chronic myeloid leukaemia during deep molecular response by genomic PCR: a traffic light stratification model with impact on treatment-free remission. Leukemia. 2020;34(8):2113-24.
6. Gabert J, Beillard E, Velden VHJvd, Bi W, Grimwade D, Pallisgaard N, et al. Standardization and quality control studies of 'real-time' quantitative reverse transcriptase polymerase chain reaction of fusion gene transcripts for residual disease detection in leukemia – A Europe Against Cancer Program. Leukemia. 2003;17(12):2318-57.
7. Cross NCP, White HE, Colomer D, Ehrencrona H, Foroni L, Gottardi E, et al. Laboratory recommendations for scoring deep molecular responses following treatment for chronic myeloid leukemia. Leukemia. 2015;29(5):999-1003.
8. Glauche I, Kuhn M, Baldow C, Schulze P, Rothe T, Liebscher H, et al. Quantitative prediction of long-term molecular response in TKI-treated CML – Lessons from an imatinib versus dasatinib comparison. Sci Rep. 2018;8(1):12330.
9. Schafer V, White HE, Gerrard G, Mobius S, Saussele S, Franke GN, et al. Assessment of individual molecular response in chronic myeloid leukemia patients with atypical BCR-ABL1 fusion transcripts: recommendations by the EUTOS cooperative network. J Cancer Res Clin Oncol. 2021.
10. Team RC. R: A language and environment for statistical computing. Vienna, Austria: R Foundation for Statistical Computing; 2020.
